# Draft Genome Sequence of *Bacillus subtilis* AS2, a Heavy Crude Oil-Degrading and Biosurfactant-Producing Bacterium Isolated from a Soil Sample

**DOI:** 10.1128/genomeA.00969-17

**Published:** 2017-09-28

**Authors:** Abdullah Al-Sayegh, Yahya Al-Wahaibi, Sanket Joshi, Saif Al-Bahry, Abdulkadir Elshafie, Ali Al-Bemani

**Affiliations:** a Department of Petroleum and Chemical Engineering, College of Engineering, Sultan Qaboos University, Muscat, Oman; b Central Analytical and Applied Research Unit, College of Science, Sultan Qaboos University, Muscat, Oman; c Department of Biology, College of Science, Sultan Qaboos University, Muscat, Oman

## Abstract

Here, we report the draft genome sequence of *Bacillus subtilis* AS2 that was isolated from heavy crude oil-contaminated soil samples from sludge pits of an Omani heavy-oil field. *B. subtilis* AS2 was able to biodegrade heavy crude oil and produce biosurfactant. In order to provide a better understanding of the biodegradation mechanism and biosynthesis of metabolites, the *B. subtilis* AS2 genome was sequenced and compared to those of other *B. subtilis* strains.

## GENOME ANNOUNCEMENT

Microbial enhanced oil recovery (MEOR) represents the use of bacteria to extract remaining oil volumes from depleting petroleum reservoirs by means of utilizing microbial products. The general bacterial mechanisms for accessing petroleum hydrophobic substrates are interfacial accession by direct contact of the cell, with the hydrocarbon and biosurfactant-mediated accession by cell contact with emulsified hydrocarbons (1). *Bacillus* bacteria are used in many processes due to their physiologic characteristics and ability to produce various metabolites (2). Fifteen different strains of *Bacillus* were isolated from heavy crude oil (HCO)-contaminated soil samples (3). *Bacillus subtilis* strain AS2 was able to degrade HCO and produce biosurfactant in different minimal salt media. The AS2 genome was sequenced and compared to those of *B. subtilis* subsp. *subtilis* strain 168 (BGSC 1A700) (4), *B. subtilis* subsp. *subtilis* ATCC 6051-HGW (5), *B. subtilis* subsp. *subtilis* BSP1 (6), *B. subtilis* subsp. *subtilis* RO-NN-1 (7), and *B. subtilis* subsp. *spizizenii* TU-B-10 (7).

The DNA was extracted by using the UltraClean soil DNA isolation kit (Mo Bio Laboratories). A paired-end sequencing library was prepared using the NEBNext Ultra DNA library preparation kit. Ligated products were purified using Ampure XP beads. The product was PCR amplified as described in the kit protocol. The amplified library was analyzed in a Bioanalyzer 2100 (Agilent Technologies) using a high-sensitivity (HS) DNA chip per the manufacturer’s instructions. The library was loaded onto an Illumina platform (2 × 150-bp read length) for cluster generation and sequencing. *De novo* assembly of high-quality paired-end (PE) reads was accomplished using Velvet v1.2.10 (8), which provided the best assembly at a k-mer value of 121. The resulting assembly generated 21 scaffolds, 4,042,051-bp genome size including gaps, 43.9% G+C content, 82 tRNAs, and 8 rRNAs. A total of 4,027 putative coding sequences (CDS) were identified using the Prodigal tool v2.6.1 (9). CDS were annotated by BLASTx search against the NR database with cutoff E values of 10^−5^. Ortholog assignment and mapping of CDS to the biological pathways were performed using the KEGG automatic annotation server (KAAS) (10). All the CDS were compared against the KEGG database using BLASTx with a threshold bit-score value of 60 (default).

With respect to crude oil and petroleum hydrocarbon biodegradation, AS2 exhibited two degradation genes, *pcaC* (benzoate) and *atoD* (aminobenzoate), which were not found in strain 168. The *pcaC* gene (benzoate) was not found in TU-B-10. There were no differences between AS2 and BSP1 or between AS2 and 6051-HGW. RO-NN-1 and TU-B-10 had nitrotoluene degradation gene K10678 *nfsA*, whereas AS2 and BSP1 did not.

With respect to biosurfactant biosynthesis, AS2 had surfactin family lipopeptide synthetase A, B, and C and iturin family lipopeptide synthetase A, B, and C. BSP1 and TU-B-10 had surfactin family lipopeptide synthetase B and C and iturin family lipopeptide synthetase A, B, and C. RO-NN-1 had surfactin family lipopeptide synthetase B and C and fengycin family lipopeptide synthetase A, B, C, D, and E.

### Accession number(s).

This whole-genome shotgun project has been deposited at DDBJ/ENA/GenBank under the accession number MUXL00000000. The version described in this paper is version MUXL01000000.

## References

[1] Wentzel A, Ellingsen TE, Kotlar H-K, Zotchev SB, Throne-Holst M 2007 Bacterial metabolism of long-chain *n*-alkanes. Appl Microbiol Biotechnol 76:1209–1221. doi:10.1007/s00253-007-1119-1. 17673997

[2] Logan NA, De Vos P 2015, Bacillus. *In* Whitman WB (ed), Bergey’s manual of systematics of archaea and bacteria. John Wiley & Sons, Hoboken, NJ. doi:10.1002/9781118960608.gbm00530.

[3] Al-Sayegh A, Al-Wahaibi Y, Al-Bahry S, Elshafie A, Al-Bemani A, Joshi S 2015 Microbial enhanced heavy crude oil recovery through biodegradation using bacterial isolates from an Omani oil field. Microb Cell Fact 14:141. doi:10.1186/s12934-015-0330-5. 26377922 PMC4573931

[4] Kunst F, Ogasawara N, Moszer I, Albertini AM, Alloni G, Azevedo V, Bertero MG, Bessières P, Bolotin A, Borchert S, Borriss R, Boursier L, Brans A, Braun M, Brignell SC, Bron S, Brouillet S, Bruschi CV, Caldwell B, Capuano V, Carter NM, Choi SK, Cordani JJ, Connerton IF, Cummings NJ, Daniel RA, Denziot F, Devine KM, Düsterhöft A, Ehrlich SD, Emmerson PT, Entian KD, Errington J, Fabret C, Ferrari E, Foulger D, Fritz C, Fujita M, Fujita Y, Fuma S, Galizzi A, Galleron N, Ghim SY, Glaser P, Goffeau A, Golightly EJ, Grandi G, Guiseppi G, Guy BJ, Haga K, Haiech J, Harwood CR, Henaut A, Hilbert H, Holsappel S, Hosono S, Hullo MF, Itaya M, Jones L, Joris B, Karamata D, Kasahara Y, Klaerr-Blanchard M, Klein C, Kobayashi Y, Koetter P, Koningstein G, Krogh S, Kumano M, Kurita K, Lapidus A, Lardinois S, Lauber J, Lazarevic V, Lee SM, Levine A, Liu H, Masuda S, Mauel C, Medigue C, Medina N, Mellado RP, Mizuno M, Moestl D, Nakai S, Noback M, Noone D, O’Reilly M, Ogawa K, Ogiwara A, Oudega B, Park SH, Parro V, Pohl TM, Portetelle D, Porwollik S, Prescott AM, Presecan E, Pujic P, Purnelle B, Rapoport G, Rey M, Reynolds S, Rieger M, Rivolta C, Rocha E, Roche B, Rose M, Sadaie Y, Sato T, Scanlan E, Schleich S, Schroeter R, Scoffone F, Sekiguchi J, Sekowska A, Seror SJ, Serror P, Shin BS, Soldo B, Sorokin A, Tacconi E, Takagi T, Takahashi H, Takemaru K, Takeuchi M, Tamakoshi A, Tanaka T, Terpstra P, Tognoni A, Tosato V, Uchiyama S, Vandenbol M, Vannier F, Vassarotti A, Viari A, Wambutt R, Wedler E, Wedler H, Weitzenegger T, Winters P, Wipat A, Yamamoto H, Yamane K, Yasumoto K, Yata K, Yoshida K, Yoshikawa HF, Zumstein E, Yoshikawa H 1997 The complete genome sequence of the Gram-positive bacterium *Bacillus subtilis*. Nature 390:249–256. doi:10.1038/36786. 9384377

[5] Kabisch J, Thürmer A, Hübel T, Popper L, Daniel R, Schweder T 2013 Characterization and optimization of *Bacillus subtilis* ATCC 6051 as an expression host. J Biotechnol 163:97–104. doi:10.1016/j.jbiotec.2012.06.034. 22789474

[6] Schyns G, Serra CR, Lapointe T, Pereira-Leal JB, Potot S, Fickers P, Perkins JB, Wyss M, Henriques AO 2013 Genome of a gut strain of *Bacillus subtilis*. Genome Announc 1(1):e00184-12. doi:10.1128/genomeA.00184-12. PMC3569322 23409263

[7] Earl AM, Eppinger M, Fricke WF, Rosovitz MJ, Rasko DA, Daugherty S, Losick R, Kolter R, Ravel J 2012 Whole-genome sequences of *Bacillus subtilis* and close relatives. J Bacteriol 194:2378–2379. doi:10.1128/JB.05675-11. 22493193 PMC3347079

[8] Zerbino DR, Birney E 2008 Velvet: algorithms for *de novo* short read assembly using de Bruijn graphs. Genome Res 18:821–829. doi:10.1101/gr.074492.107. 18349386 PMC2336801

[9] Hyatt D, Chen G-L, LoCascio PF, Land ML, Larimer FW, Hauser LJ 2010 Prodigal: prokaryotic gene recognition and translation initiation site identification. BMC Bioinformatics 11:119. doi:10.1186/1471-2105-11-119. 20211023 PMC2848648

[10] Moriya Y, Itoh M, Okuda S, Yoshizawa AC, Kanehisa M 2007 KAAS: an automatic genome annotation and pathway reconstruction server. Nucleic Acids Res 35:W182–W185. doi:10.1093/nar/gkm321. 17526522 PMC1933193

